# Application of Silver-Loaded Composite Track-Etched Membranes for Photocatalytic Decomposition of Methylene Blue under Visible Light

**DOI:** 10.3390/membranes11010060

**Published:** 2021-01-15

**Authors:** Anastassiya A. Mashentseva, Murat Barsbay, Nurgulim A. Aimanova, Maxim V. Zdorovets

**Affiliations:** 1 The Institute of Nuclear Physics of the Republic of Kazakhstan, Ibragimov Str., 1, Almaty 050032, Kazakhstan; nurgulim.a.a@gmail.com (N.A.A.); mzdorovets@gmail.com (M.V.Z.); 2 Department of Chemistry, Hacettepe University, 06800 Beytepe, Ankara, Turkey; mbarsbay@hacettepe.edu.tr; 3 Engineering Profile Laboratory, L.N. Gumilyov Eurasian National University, Satpaev Str., 5, Nur-Sultan 010008, Kazakhstan; 4 Department of Intelligent Information Technologies, The Ural Federal University Named after the First President of Russia B. N. Yeltsin, Mira Str. 19, 620002 Yekaterinburg, Russia

**Keywords:** track-etched composite membranes, electroless template synthesis, composite nanocatalyst, silver microtubes, methylene blue decomposition

## Abstract

In this study, the use of composite track-etched membranes (TeMs) based on polyethylene terephthalate (PET) and electrolessly deposited silver microtubes (MTs) for the decomposition of toxic phenothiazine cationic dye, methylene blue (MB), under visible light was investigated. The structure and composition of the composite membranes were elucidated by scanning electron microscopy, energy dispersive spectroscopy, and X-ray diffraction technique. Under visible light irradiation, composite membrane with embedded silver MTs (Ag/PET) displayed high photocatalytic efficiency. The effects of various parameters such as initial dye concentration, temperature, and sample exposure time on the photocatalytic degradation process were studied. The decomposition reaction of MB was found to follow the Langmuir–Hinshelwood mechanism and a pseudo-first-order kinetic model. The degradation kinetics of MB accelerated with increasing temperature and activation energy, *E*_a_, was calculated to be 20.6 kJ/mol. The reusability of the catalyst was also investigated for 11 consecutive runs without any activation and regeneration procedures. The Ag/PET composite performed at high degradation efficiency of over 68% after 11 consecutive uses.

## 1. Introduction

Currently, more than 100,000 synthetic dyes are commercially available and are widely used in industry. They represent one of the largest groups of toxic and carcinogenic organic compounds, and almost 20% of the world’s water pollution is caused by dyes [1,2]. Extremely high reactivity and excessive toxicity of dyes in high doses (especially for diazo-, direct, and basic water-soluble dyes) pose severe environmental hazards [3]. World Health Organization cites these compounds among the main causes of water pollution worldwide [4,5]. Of the toxic water pollutants identified (chemical dyes, pesticides, paints, and solvents), about 72 are dye compounds, and 30 of them are non-degradable. [5]. The restoration of dyes in the human intestinal environment has previously been shown to lead to the formation of highly toxic amines [6,7]. Also, harmful carcinogenic species such as aromatic compounds and benzidine are used to produce a range of dyes, which makes them extremely dangerous to human health [4,8]. The development of high-efficiency catalytic systems for the decomposition of dyes is a very attractive area in the processing industry. In the last decade, there has been a lot of research focusing on the use of nanomaterial-based photocatalysts in decomposition of dye pollutants [9,10,11,12].

Commonly applied water treatment methods, such as adsorption or coagulation, only concentrate or move the pollutants into other materials, but the contaminants still exist and are not fully cleared off [13]. High operating costs and the potential to generate harmful secondary pollutants in the environment are problems associated with other conventional methods of water treatment, such as sedimentation, filtration, membrane processes and chemical technologies [14,15]. The most commonly used disinfection process is chlorination, but the by-products of chlorine-based disinfection are mutagenic and carcinogenic for human health [15]. Among a wide range of water treatment approaches, the most promising group is the advanced oxidation processes (AOP) [16,17]. A special place in this group is occupied by heterogeneous photocatalysis, which demonstrated its effectiveness in degrading a wide range of dyes into readily biodegradable compounds and eventually mineralized them into innocuous carbon dioxide and water. Semiconductors such as TiO_2_, ZnO, Fe_2_O_3_, CdS, and ZnS are generally used as photocatalysts for industrial use [18,19,20,21,22]. There are also studies on the use of various metal nanoparticles for photocatalytic degradation of dyes [12,20,23,24]. The development of highly effective and inexpensive catalysts for the decomposition of synthetic dyes is one of the focus areas of the world’s leading research groups.

The use of porous photocatalytic membranes offers the most attractive approach, provided they can be designed to offer a combination of high specific surface area and excellent permeability [25,26]. A wide range of different scaffold types have been investigated for the creation of such membrane photoreactors [26]. However, the prospects of applying track-etched membranes (TeMs) as a support for the photocatalytic membrane reactor have not been adequately explored. TeMs with deposited nano- or microtubes (MTs) are among the promising composite functional materials with hollow nanostructures embedded in polymer templates [27,28]. They are successfully used in nanocatalysis [29,30,31,32], nanosensors [33,34], and radiation material science [35,36]. In our previous studies, silver-loaded composite TeMs were successfully investigated as efficient catalysts in hydrogen peroxide decomposition and p-nitrophenol reduction [37,38,39]. In all these studies, composite membranes could withstand no more than 5–6 test cycles without additional activation and regeneration procedures.

The aim of this study was to evaluate the catalytic performance of composite TeMs with electrolessly deposited silver microtubes (MTs) in the decomposition of a model dye, methylene blue (MB), under visible light. It has previously been shown that nanosized silver is an effective catalyst for a variety of reactions [37,40], including photocatalytic decomposition of some organic pollutants [41,42]. Considering the porosity-induced high surface area of the TeMs and the high catalytic activity of silver nanoparticles, it is believed that the composite membranes developed will stand out as promising alternatives in water treatment. Catalysts deposited on a porous substrate have several advantages compared to non-supported analogues: Catalytically active nanoparticles or nanopowders must be carefully separated from the solution by filtration, precipitation, or centrifugation, which are rather laborious and uneconomical procedures [43]. Flexible TeMs with deposited nanotubes/nanoparticles can be easily removed after reaction and reused without any additional activation procedure.

## 2. Materials and Methods

### 2.1. Chemical Reagents

Silver nitrate (AgNO_3_), tin(II) chloride (SnCl_2_), pyridine, methylene blue, sodium potassium tartrate (Sigma-Aldrich, St. Louis, MO, USA), and all other chemicals were analytical grade and used without further purification. All aqueous solutions were prepared using deionized water (18.2 MΩ/cm, Aquilon D-301).

### 2.2. Synthesis of Ag/PET Composite Catalyst

A 12.0-μm-thick polyethylene terephthalate (PET) film irradiated with accelerated krypton (energy 1.75 MeV/nucleon, pores’ density of 4 × 10^7^ pores/cm^2^) was used as the template membrane. After chemical etching (2.2 M NaOH, 358 K), the pore size of the TeM was found to be 430 ± 15 nm.

Prior to electroless plating, the surface of the PET template was sequentially treated in solutions of sensitization (50 g/L SnCl_2_, 60 mL/L HCl (37%) for 15 min) and activation (59 mM AgNO_3_, 230 mM NH_3_ in water for 3 min). The sample was then immersed in an aqueous solution containing 17 mM AgNO_3_, 120 mM potassium tartrate, and 50 mM pyridine as complexing additive [37,44]. Deposition was carried out in a constant temperature water bath (275 ± 1 K) for the required time.

### 2.3. Characterization

The morphology and chemical composition of the composite membrane with embedded silver MTs (Ag/PET) were examined by scanning electron microscopy (SEM, JFC-7500F, JEOL Ltd., Tokyo, Japan) and energy dispersive X-ray spectroscopy (EDX) using a Hitachi TM3030 microscope equipped with a Bruker XFlash MIN SVE microanalysis system (Hitachi Ltd., Chiyoda, Tokyo, Japan) (accelerating voltage-15 keV). In order to show that the nanochannels running along the cross section of the PET membrane were completely filled with silver, the PET template was dissolved in 1,1,1,3,3,3-hexafluoro-2-propanol/chloroform solution (1:9 *v*/*v*) for 2 h [45]. SEM images of the silver MTs were recorded after the release from the template. 

X-ray diffraction (XRD) measurements of the released silver MTs were obtained on a D8 Advance diffractometer (Bruker, Karlsruhe, Germany). X-ray was generated at 40 mA and 40 kV and the scanning position ranged from 20–140° 2(θ). The crystal grain sizes were calculated using the Scherrer equation [46]. 

The pore sizes of the PET track-etched membrane (TeM) and the wall thickness of the obtained MTs were determined using the Hagen–Poiseuille equation (Equation (1)), described in detail elsewhere [47].

(1) $$Q=\frac{8\pi }{3MRT}\sqrt{\frac{nr^{3}\Delta p}{l}}$$

where Δ*p* is the pressure difference, MPa; *M* is the molecular mass of the gas (compressed air), dyn × cm^−2^; *R* is the universal gas constant, erg/(mol × K); *n* is the number of microtubes per square centimeter of membrane area (template pore density); *l* is the membrane thickness, cm; and *T* is temperature, K.

### 2.4. Photocatalytic Activity

Methylene blue (MB) was used as a model dye to examine the photocatalytic activity of as-prepared composite membranes under visible light irradiation. All experiments were carried out in 200-mL double-wall glassware under visible light using a 500-W halogen lamp with UV cutoff filter (Econur, Vito Electric). The distance from the light source to the working solution was 15 cm. Light intensity was controlled by AS803 digital lux meter (Smart sensor, China) and was found to be 7500 Lm. In a typical procedure (scheme of laboratory setup used is presented in the Figure S1 (Figure S1 can be found in the Supplementary Materials), a 2 × 2 cm Ag/PET composite membrane is immersed in 100 mL of MB solution (concentration is varied in the range of 0.1–5.0 mg/L) and stirred in the dark for 1 h to achieve adsorption–desorption equilibrium between organic dye and catalyst. After starting to irradiate with visible light, a 2-mL aliquot was taken every 5–10 min from the reaction mixture and its absorbance was measured using a Specord–250 spectrophotometer (Analytic Jena, Jena, Germany) in the wavelength range of 200–800 nm. According to the Beer–Lambert law, the concentration of MB is directly proportional to its absorbance. Thus, the degradation rate of MB was calculated based on its characteristic peak at 664 nm using the following equation [48]:

(2) $$D=\frac{C_{0}-C}{C_{0}}\times 100\%=\frac{A_{0}-A}{A_{0}}\times 100\%$$

where A_0_ is the initial absorbance of MB solution at 664 nm before loading the catalyst, A is the absorbance at 664 nm at different time intervals, and C_0_ is the concentration of feed solution.

## 3. Results and Discussions

### 3.1. Ag/PET Composite Synthesis

**Figure 1:**
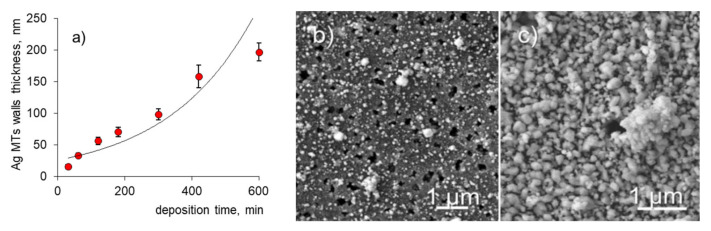
The change in wall thickness of silver microtubes (MTs) depending on the deposition time (**a**) and SEM images of the composites’ surface after 30 min (**b**) and 7 h of deposition (**c**).

**Figure 2:**
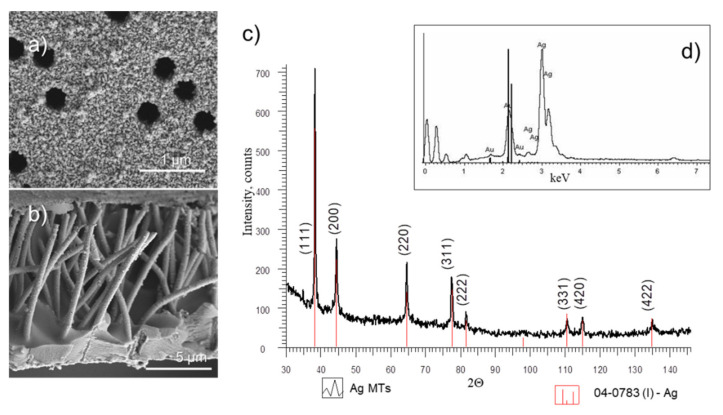
SEM images of the surface (**a**) and released individual Ag MTs (**b**), X-ray diffraction pattern (**c**), and energy dispersive X-ray spectrum (**d**) of the released silver MTs after removal of the polymer template.

A series of plating experiments were conducted to evaluate the dependence of the wall thickness of the silver MTs on the deposition time (Figure 1). The deposition rate, R, of the electroless silver plating was expressed as weight gain per 1 cm^2^ of PET TeMs during the deposition process and was found to be 1.1 ± 0.01 mg/cm^2^ × h at 275 K for all prepared samples. When the deposition time was more than 5 h (e.g., 7 h, Figure 1c), both surfaces of the membrane were completely covered with large silver lumps and, as a result, the pores of the membrane were completely closed. The use of composites obtained at a deposition time of 0.5–1.0 h was rather difficult due to their unstable mechanical properties and the active removal of metallic nanoparticles from the surface during catalytic tests under vigorous stirring [30,49]. The optimal deposition time was determined as 5 h, when the highest amount of loaded silver was obtained without blocking the pores. As can be seen in Figure 2a, a uniform deposition of silver was obtained without closing the pores over the entire surface of the polymer template after a 5-h deposition period.

To show that the nanochannels running along the cross section of the PET membrane were completely filled with silver, the PET template was dissolved and the released Ag MTs were characterized by SEM and XRD (Figure 2).

After dissolving of the PET matrix, the X-ray diffraction pattern of the silver MTs (Figure 2c) clearly showed the characteristic (111), (200), (220), and (311) silver planes at 2θ angles of 38.16°, 44.35°, 64.55°, and 77.45°, respectively. The value of the face-centered cubic (fcc) cell constant a = (4.0838–0.035) Å was in full agreement with the reference data of a = 4.086 nm (Joint Committee on Powder Diffraction Standards (JCPDS) card 4-0783). The crystallite size obtained from the full width at half maximum (FWHM) values was approximately 24.6 ± 5 nm. From the porometry data, the inner diameter and wall thickness of the MTs were found as 319.0 and 63.0 nm, respectively. The silver loading in the composite was calculated gravimetrically as 0.6 ± 0.01 mg/cm^2^.

The chemical composition of the synthesized microstructures according to the energy dispersive X-ray spectrum (Figure 2d) was represented only by the silver phase. The presence of gold phase (2.5–2.8%) in the EDX spectrum arose due to the sample preparation process performed by magnetron sputtering prior to analysis.

### 3.2. Catalytic Activity Results

**Figure 3:**
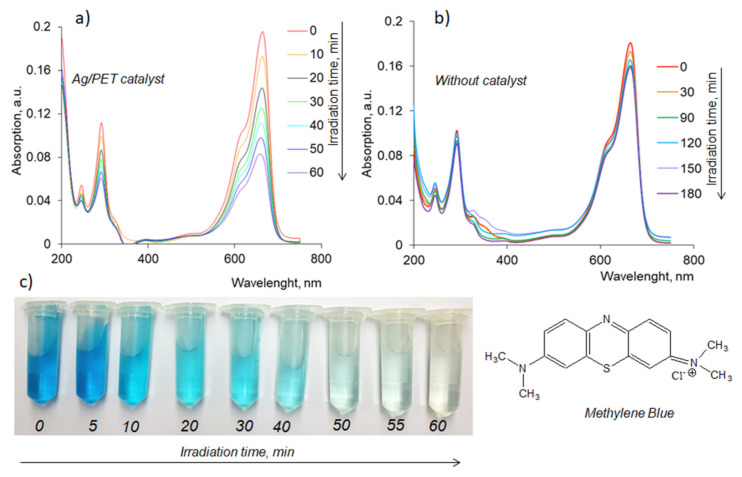
Typical absorption spectra for the decomposition reaction of methylene blue (MB) (0.5 mg/L) in the presence of 2 × 2 cm Ag/Polyethylene terephthalate (Ag/PET) composite membrane (**a**) and without any catalyst (**b**). Visual observation of color change from blue to colorless indicating the degradation of MB (0.1 mg/L) by the composite catalyst at different time intervals (**c**).

MB is a toxic phenothiazine cationic dye at high doses [50] used on an industrial scale for dyeing paper, cotton, wool, etc. [51]. MB decomposition is one of the most popular test reactions used as a reference in determining the photoactivity of semiconductor and nanoscale materials [52,53,54,55]. In this study, the effects of dye concentration, sample exposure time, and temperature on the catalytic activity of composite Ag/PET TeMs along with the stability of catalysts’ properties were investigated. Absorption spectra of aqueous MB solutions measured in the presence and absence of the composite catalyst at different times are presented in Figure 3a,b.

After 60 min of exposure to visible light, the intensity of the characteristic peak of MB at 664 nm decreased significantly in the presence of Ag/PET composite (Figure 3a). Significant discoloration of solution due to MB decomposition could be easily seen with the naked eye (Figure 3c). On the other hand, similar tests performed without the catalyst do not show significant changes in absorbance even after 180 min of visible light exposure, and the maximum degree of dye decomposition (D, %) was calculated to be only 4.4%.

#### 3.2.1. Effect of Initial Dye Concentration

**Figure 4:**
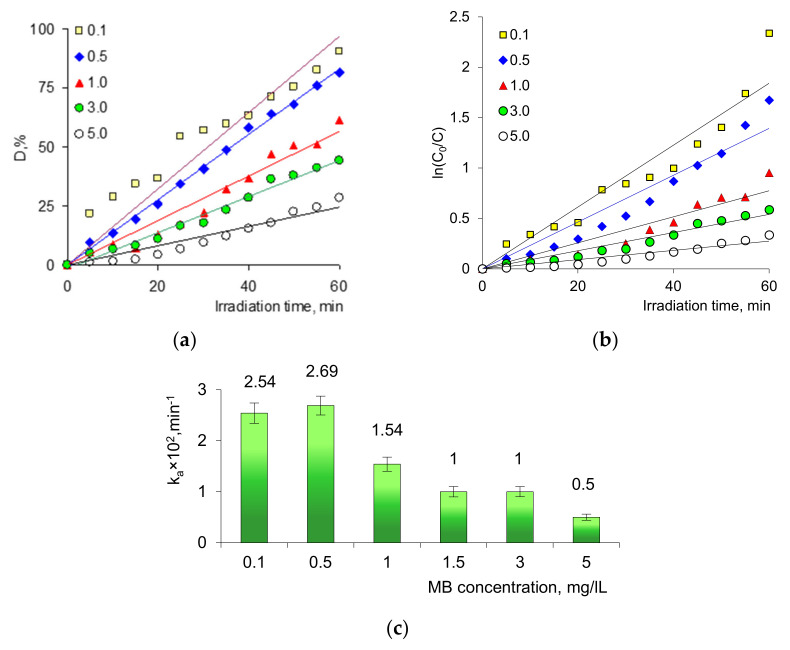
The change of degree of dye decomposition (D, %) achieved at various MB feed concentrations in the presence of Ag/PET composite (**a**), variation of natural logarithm of normalized concentration (ln(C_0_/C)) as a function of visible light irradiation time (**b**). and change of the apparent rate constant $k_{a}$ values at different MB feed concentrations (**c**).

The effect of initial dye concentration on degradation efficiency under visible light irradiation was investigated by changing the feed concentration of MB solutions in the range of 0.1–5.0 mg/L. In all experiments, the same amount of catalyst (2 × 2 cm, 2.4 mg of loaded silver) was employed at a temperature of 331 K and a pH value of 6.5. Figure 4a shows the change in the degree of dye decomposition (D, %) under a 500-W halogen lamp over different periods of sample exposure up to 60 min, depending on the MB feed concentration. As can be seen, the value of D depended on the initial MB concentration. Almost complete degradation (>85%) occurred in 60 min when the initial concentration of MB was low, e.g., 0.1 and 0.5 g/L. At a concentration of 1.0 and 5.0 mg/L, the reaction mixture had to be irradiated for 155 and 370 min, respectively, to achieve more than 90% MB decomposition. The decomposition reaction of MB followed the Langmuir–Hinshelwood mechanism [56,57] and a pseudo-first-order kinetics model, which allows one to calculate the apparent rate constant from the change in the dye concentration [58]:

(3) $$\ln\left(\frac{C_{0}}{C}\right)=k_{a}t$$

where C_0_ is the initial concentration of the dye (mg/L), C is the concentration of the dye at the time t, t is the irradiation time (min), and $k_{a}$ is the reaction rate constant (min^−1^).

Kinetic curves for visible light-induced decomposition of aqueous solutions of MB in the concentration range of 0.1–5.0 mg/L in the presence of Ag/PET composite (2 × 2 cm) are shown in Figure 4b. As can be seen from the curves, they followed pseudo-first-order kinetics, as confirmed by the linear character of the t versus natural logarithm of normalized concentration ln(C_0_/C) plots. The apparent rate constants calculated from these curves are presented in Figure 4c. The equations of the regression line and corresponding coefficients of determination R^2^ are presented in Table S1 (Table S1 can be found in the Supplementary Materials).

Based on the data obtained, it can be noted that an increase in dye concentration caused a decrease in decomposition efficiency for two reasons. First, the concentrated solution acquired a more intense color that prevented the penetration of radiation onto the catalyst surface [59]. Second, as other experimental conditions were the same, the ratio of the number of hydroxyl radicals, •OH, to the number of dye molecules decreased with increasing concentration. Therefore, the reaction rate decreased with increasing initial dye concentration, as can be seen from the $k_{a}$ values presented in Figure 4c [60].

#### 3.2.2. Effect of Temperature on the Decomposition of MB

**Figure 5:**
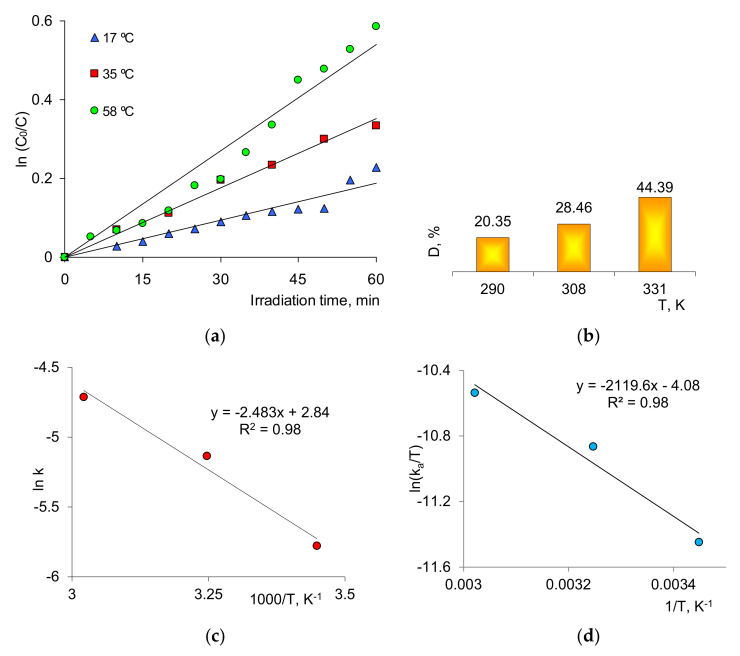
Efficiency of decomposition of MB (1.0 mg/L) in the presence of composite Ag/PET TeM at various temperatures (**a**,**b**). Arrhenius graph for determining Ea (**c**) and the Eyring plot for determining activation entropy (Δ*S*^≠^) and activation enthalpy (ΔH^≠^) (**d**).

The effect of temperature on the degradation efficiency in the presence of the composite catalysts was studied in the temperature range of 290–331 K. Figure 5a shows the change in ln(C_0_/C) depending on the irradiation time and test temperature (3.0 mg/L). Activation energy (Ea) was determined as 20.6 kJ/mol using the Arrhenius plot (Figure 5c). The data obtained were consistent with the results of previous studies where the Ea was reported as 27.1 and 25.7 kJ/mol for 0.1 g Ag-TiO_2_ [59] and 0.1 g of 3% Ag/Al_2_O_3_ [61] composites, respectively. As an advantage, the results in our study indicated a lower activation energy. The apparent rate constant, $k_{a}$, increased from 0.31 to 0.9 min^−1^ as the temperature rose from 290 to 331 K. Figure 5b shows the degree of decomposition of MB (1.0 mg/L) at various temperatures after 60 min of exposure. We concluded that the reaction temperature accelerated the decomposition of MB by increasing the mobility of the reactive radical species and the desorption of colorless decomposition products of MB from the surface of catalyst.

The Eyring equation [62,63] was used to calculate activation enthalpy (Δ*H*^≠^; kJ/mol) and entropy (Δ*S*^≠^; J mol/K) from the slope and intercept of ln(*k_a_/T*) versus 1/*T* line (Figure 5d), respectively, Equation (4):

(4) $$\ln\left(\frac{k_{a}}{T}\right)=\ln\left(\frac{k_{B}}{h}\right)+\left(\frac{\Delta S^{\neq }}{R}\right)-\left(\frac{\Delta H^{\neq }}{RT}\right)$$

where $k_{B}\;$ and *h* are the Boltzmann and Planck constants, respectively. The enthalpy change was calculated as 18.07 kJ/mol and the entropy change as −0.165 kJ/molK. Given the positive ΔH^≠^ and negative ΔS^≠^ values, it appeared that endothermic interactions and a decrease in entropy occurred at the solid–liquid interface during the decomposition process of MB on the surface of Ag/PET composite TeMs [62].

#### 3.2.3. The Stability of the Properties of the Composite Catalyst

**Figure 6:**
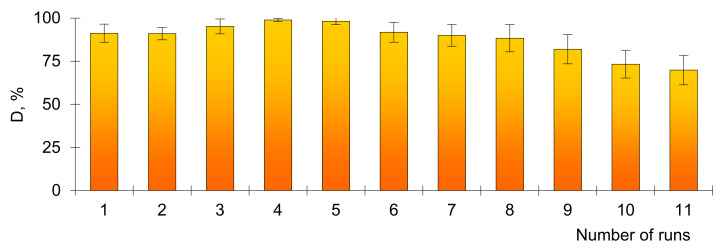
Change in the degradation efficiency D of MB (0.1 mg/L) dye for several consecutive runs.

Besides catalytic activity, structural stability and reusability are critically important for practical dye decomposition applications. In this study, 11 consecutive test cycles (Figure 6) were performed on the decomposition of MB (0.1 mg/L) to assess the stability of the properties of the studied composites. (All tests were carried out without any activation and regeneration procedure).

As can be seen from the data in Figure 6, after several test cycles, the decomposition efficiency of MB remained almost unchanged. The composite catalyst was still able to decompose more than 68% of MB at the 11th run, indicating excellent recyclability of the silver-loaded composite track-etched membrane as a catalyst. The excellent recyclability was attributed to the very stable presence of the silver MTs in TeM structure. Table 1 summarizes some previously published comparative results on the photocatalytic activity of various silver-based nanoscale structures employed in the decomposition of MB.

It should be noted that it was rather difficult to directly compare data of various studies, as factors, such as irradiation conditions (power and type of lamps), the amount of loaded catalyst, the dye concentration in the tests, etc., vary widely. Nevertheless, from the results presented in Table 1, it can be easily stated that the catalysts obtained in this study performed at a level similar to the literature, with less catalyst use. Considering the ease of reuse of the composite TeMs, which does not require additional steps such as filtration, activation, sedimentation, or centrifugation before recycling, it can be said that the catalyst developed in this study was very advantageous and exhibited high industrial use potential. The results obtained in this study not only offer the opportunity to use the silver-loaded composite TeMs in the decomposition of MB, but also show promise for a possible use of this photoactive catalyst in the degradation of other dyes.

## 4. Conclusions

Porous composite catalyst, based on PET track-etched membranes and silver MTs, was successfully produced by electroless template deposition approach. Optimal silver plating conditions (plating time) were determined, and optimal plating time was found as 5 h using data from SEM and porometry. Electroless deposition in polymer template (pore size of 430 ± 15 nm) was applied to produce composite TeMs with Ag MTs with wall thickness and inner diameter of 63.0 and 319 nm, respectively. XRD, SEM, and EDX analysis confirmed the chemical structure and morphology of silver MTs.

Synthesized Ag/PET composites exhibited significant photocatalytic activity in the degradation of MB under visible light. In the presence of the composite TeMs, the dye decomposition kinetics followed the Langmuir–Hinshelwood mechanism and was dependent on the dye concentration. It was found that endothermic interactions and a decrease in entropy occurred during the decomposition of MB at the solid–liquid interface. Recyclability test was carried out for up to 11 runs and the results showed excellent reusability. The influence of temperature in the range of 17–58 °C was studied and the activation energy was calculated as 20.6 kJ/mol.

Given its significant catalytic activity and ease of use, this economical and environmentally friendly composite catalyst could offer wastewater treatment potential for degradation of organic dye contaminants such as the model dye (MB) investigated in this study.

## Figures and Tables

**Table 1:** Some important features and photocatalytic activity of silver-based nanostructures employed in the MB decomposition.

Loaded Catalyst	mg	Nanocatalyst Test Conditions			D, %	Reference
		Light Source	Exposure Time, min	MB, mg/L		
Ag NPs	10.0	sunlight	120	10.0	4.7	[64]
Ag/ZnO	5.0	sunlight	20	3.2	75.3	[65]
Ag/Al_2_O_3_	100.0	sunlight	120	100.0	100.0	[61]
Ag/Cu_2_O	20.0	500 W halogen lamp	120	10.0	96.5	[66]
Ag-doped ZnO nanorods	100.0	100 W halogen lamp	120	6.0	76.0	[67]
Ag/MoS_2_	1000	100 W halogen lamp	80	1.0	77.9	[68]
Ag/TiO_2_	10.0	500 W halogen lamp	200	2.0	75.0	[69]
rGO/Ag/Fe-doped TiO_2_	10	35 W Xe arc lamp	150	20.0	95.3	[70]
Ag/PET TeMs	2.4	500 W halogen lamp	60	1.0	61.4	This work

## Data Availability

All data generated or analyzed during this study are included in this published article (and its supplementary information files).

## Supplementary Materials

The following are available online at https://www.mdpi.com/2077-0375/11/1/60/s1, Figure S1: Lab setup for the silver loaded polyethylene terephthalate (Ag/PET) composite track-etched membranes (TeMs) catalytic activity examination, Table S1: The equation of the regression line and corresponding coefficient of determinations for the methylene blue (MB) decomposition reaction in the presence of the silver loaded polyethylene terephthalate Ag/PET composite track-etched membranes (TeMs).
