# Supplementary material for: Application of Silver-Loaded Composite Track-Etched Membranes for Photocatalytic Decomposition of Methylene Blue under Visible Light
Source: Membranes (Basel). 2021 Jan 15;11(1):60. doi: 10.3390/membranes11010060 (PMC7830284; doi:10.3390/membranes11010060)
Supplement: Supplementary file 1 [file membranes-11-00060-s001.pdf]

membranes

Supplementary information

Application of Silver Loaded Composite Track-Etched Membranes for Photocatalytic Decomposition of Methylene Blue under Visible Light

Anastassiya A. Mashentseva [1,*], Murat Barsbay [2], Nurgulim A. Aimanova [1,3] and Maxim V. Zdorovets [1,3,4]

1 The Institute of Nuclear Physics of the Republic of Kazakhstan, Ibragimov str., 1, 050032, Almaty, Kazakhstan; a.mashentseva@inp.kz (A.A.M.); nurgulim.a.a@gmail.com (N.A.A.); mzdorovets@gmail.com (M.V.Z.)
2 Department of Chemistry, Hacettepe University, 06800 Beytepe, Ankara, Turkey mbarsbay@hacettepe.edu.tr
3 L.N. Gumilyov Eurasian National University, Satpaev str., 5, 010008, Nur-Sultan, Kazakhstan
4 The Ural Federal University named after the first President of Russia B. N. Yeltsin, Mira str. 19, 620002 Yekaterinburg, Russia; https://urfu.ru/en/
* Correspondence: a.mashentseva@inp.kz; Tel.: +7-707-322-4399

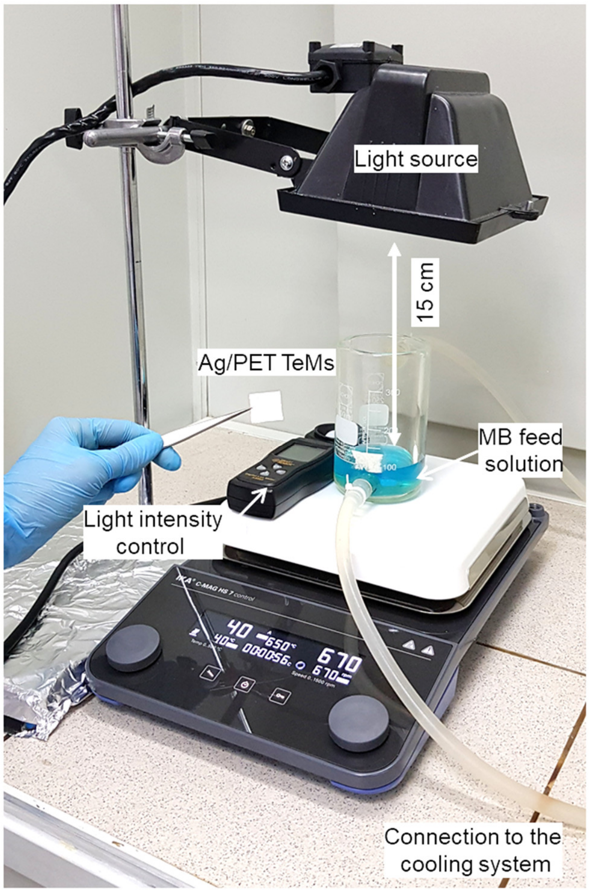

Figure S1. Lab setup for the silver loaded polyethylene terephthalate (Ag/PET) composite track-etched membranes (TeMs) catalytic activity examination.

Table S1. The equation of the regression line and corresponding coefficient of determinations for the methylene blue (MB) decomposition reaction in the presence of the silver loaded polyethylene terephthalate (Ag/PET) composite track-etched membranes (TeMs).

MB concentration, mg/l	Regression equation	Coefficients of determinations R^2
0.1	y = 0.0254x	0.94
0.5	y = 0.0269x	0.95
1.0	y = 0.0154x	0.95
3.0	y = 0.0099x	0.97
5.0	y = 0.0049x	0.98
